# Network analysis used to investigate the interplay among somatic and psychological symptoms in patients with cancer and cancer survivors: a scoping review

**DOI:** 10.1007/s11764-024-01543-0

**Published:** 2024-03-26

**Authors:** G. Elise Doppenberg-Smit, Femke Lamers, Myra E. van Linde, Annemarie M. J. Braamse, Mirjam A. G. Sprangers, Aartjan T. F. Beekman, Henk M. W. Verheul, Joost Dekker

**Affiliations:** 1 Department of Psychiatry, Amsterdam UMC, Location Vrije Universiteit Amsterdam, de Boelelaan 1117, Amsterdam, the Netherlands; 2 Amsterdam Public Health, Mental Health Program, Amsterdam, the Netherlands; 3 Cancer Centre Amsterdam, Cancer Treatment and Quality of Life, Amsterdam, the Netherlands; 4 Department of Medical Oncology, Amsterdam UMC, Location Vrije Universiteit Amsterdam, de Boelelaan 1117, Amsterdam, the Netherlands; 5 Department of Medical Psychology, Amsterdam UMC, Location University of Amsterdam, Amsterdam, the Netherlands; 6 Department of Medical Oncology, Erasmus MC, Dr. Molewaterplein 40, Rotterdam, the Netherlands

**Keywords:** Cancer, Network analysis, Symptoms, Psychological adjustment

## Abstract

**Purpose:**

Patients with cancer often experience multiple somatic and psychological symptoms. Somatic and psychological symptoms are thought to be connected and may reinforce each other. Network analysis allows examination of the interconnectedness of individual symptoms. The aim of this scoping review was to examine the current state of knowledge about the associations between somatic and psychological symptoms in patients with cancer and cancer survivors, based on network analysis.

**Methods:**

This scoping review followed the five-stage framework of Arksey and O’Malley. The literature search was conducted in May, 2023 in PubMed, APA PsycINFO, Embase Cochrane central, and CINAHL databases.

**Results:**

Thirty-two studies were included, with eleven using longitudinal data. Seventeen studies reported on the strength of the associations: somatic and psychological symptoms were associated, although associations among somatic as well as among psychological symptoms were stronger. Other findings were the association between somatic and psychological symptoms was stronger in patients experiencing more severe symptoms; associations between symptoms over time remained rather stable; and different symptoms were central in the networks, with fatigue being among the most central in half of the studies.

**Implications for Cancer Survivors:**

Although the associations among somatic symptoms and among psychological symptoms were stronger, somatic and psychological symptoms were associated, especially in patients experiencing more severe symptoms. Fatigue was among the most central symptoms, bridging the somatic and psychological domain. These findings as well as future research based on network analysis may help to untangle the complex interplay of somatic and psychological symptoms in patients with cancer.

**Supplementary Information:**

The online version contains supplementary material available at 10.1007/s11764-024-01543-0.

## Background

Patients with cancer often experience multiple symptoms related to their disease and treatment [1–3]. A distinction is generally made between somatic and psychological symptoms [4–6], which corresponds to a similar distinction between biological and psychological factors in the biopsychosocial model of health and its applications (e.g., [7–9]). Both cancer and its treatment may cause a wide range of somatic symptoms. Examples include nausea, lack of appetite, and pain [10, 11]. Cancer diagnosis and treatment lead to a process of psychological adjustment [12]; this may be accompanied by numerous psychological symptoms, such as depressive mood or anxiety [13, 14]. For some symptoms, it is less clear whether they are somatic or psychological because they may be linked to the disease and treatment as well as to the process of adaptation. Fatigue is a prime example [15–18]. A true distinction between somatic and psychological symptoms is not possible, as mind and body are not separate entities and their functioning involves shared processes [19]. Nevertheless, the distinction between somatic and psychological symptoms, with some symptoms having an undecided status, is well-established in research and clinical practice [10, 13–15].

**Fig. 1:**
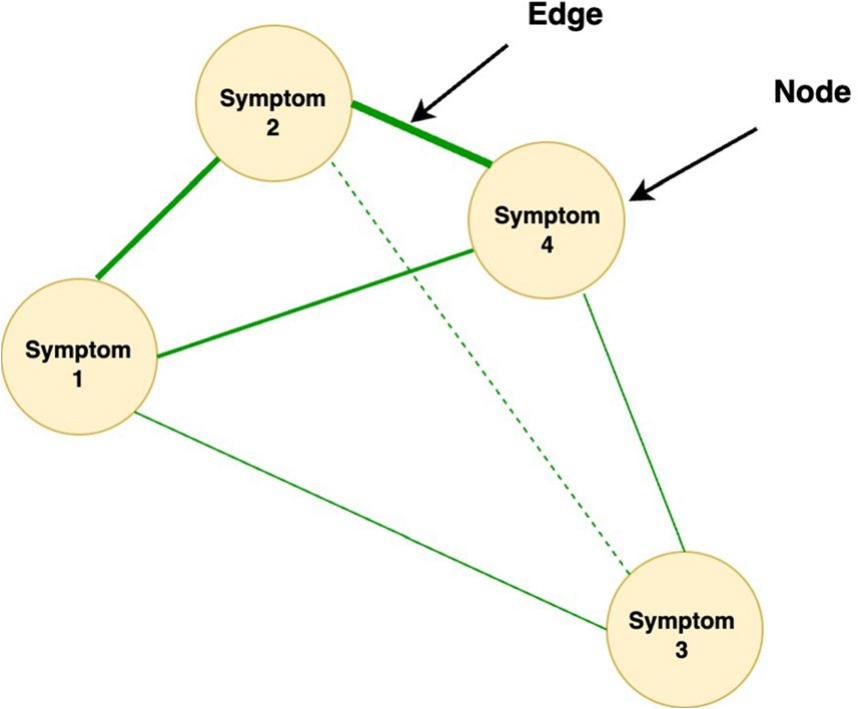
Example of symptom network. The lines are the edges (associations among symptoms), the symptoms are the nodes. When two nodes are highly correlated, the edges will be thicker. Nodes with many edges are often more centered within the network, as compared to nodes that have fewer/weaker edges

Somatic and psychological symptoms have frequently been found to be associated [15, 20–22]. However, the associations tend to be limited or are found only for certain components of mental health (e.g., depression but not distress) [23, 24]. These studies typically use latent constructs to assess somatic and psychological symptoms. In this approach, symptoms are viewed as interchangeable consequences of an underlying cause, such as cancer (for somatic symptoms) or a mental disorder (for psychological symptoms) [19]. The network theory offers a different approach to examine somatic and psychological symptoms and their association. The network theory [25] views symptoms as causally connected within a network. Network models consist of *nodes* (the variables or symptoms of interest) and *edges* (the lines between the nodes that depict the association between the nodes; see Fig. 1). In case of strong connections between symptoms in the network, activation of one symptom leads to the activation of other symptoms, resulting in cascading activation of symptoms [26]. Network analysis appears ideally suited to capture the complexity of the interplay between somatic and psychological symptoms [27]. Instead of looking for underlying causes of somatic and psychological symptoms and their association, network analysis captures the association among somatic and psychological symptoms at the level of the symptoms themselves [19]. As a result, regardless of whether a symptom (e.g., lack of appetite) is primarily due to the cancer and its treatment or to psychological adaptation, the interaction of the symptom with other symptoms can be examined directly [26]. While symptoms can often indeed be attributed primarily to cancer and its treatment or to the process of psychological adaptation, network analysis offers the opportunity to study the interaction between symptoms without assuming a particular cause of symptoms in advance. See [27] for more information on network analysis.

Research on the association between somatic and psychological symptoms in patients with cancer based on network analysis is an emerging field. An overview of the major research themes and findings in this field is not yet available. The aim of this scoping review was to provide an overview of the current state of knowledge about the association between somatic and psychological symptoms in people with cancer and cancer survivors, based on network analysis.

## Methods

A scoping review was conducted, using the five-stage framework from Arksey and O’Malley [28]. The five stages of review included the following: (1) identification of the research question; (2) identifying relevant studies; (3) study selection; (4) charting the data; (5) collating, summarizing, and reporting the results [28]. We followed the PRISMA guidelines for reporting on scoping reviews [29]. No ethics approval was required for this review.

### Identification of relevant studies

The final literature search was conducted in May 2023. The search terms focused on study population, reported symptoms, and analytic technique. For study population, both patients with cancer and cancer survivors ≥ 18 years old were included. Survivors were included because there is no sharp distinction between patients and survivors, and survivors also experience a wide range of somatic and psychological symptoms. In this review, the term “patients” can refer to patients, survivors, or both. Search terms for symptoms included both somatic and psychological symptoms that are common during or after cancer treatment, such as “nausea,” “lack of appetite,” “anxiety,” and “depression.” For analytic technique, search terms related to network analysis were included. See Appendix A for the search in PubMed.

Potential relevant articles were identified through a systematic search using a combination of operator-specific keywords in the PubMed, APA PsycINFO, Embase, Cochrane central, and CINAHL databases. References of included articles and relevant reviews were hand-searched for eligible articles.

### Study selection

Full-text articles were selected if they (1) included a sample of patients with cancer or cancer survivors, aged 18 years or older; (2) reported on the association between somatic and psychological symptoms; (3) used network analysis to examine this association; (4) were published in peer-reviewed journals; and (5) were written in English***.*** Reviews, commentaries, abstracts, and other articles that did not include data based on network analysis were excluded.

The study selection consisted of two stages. In the first stage, all articles were screened by title and abstract by one reviewer (ED). Inter-rater reliability was checked as follows. In addition to the primary reviewer ED, two independent reviewers (FL and JD) screened a random sample of 50 articles. The cut off to be met was kappa = 0.80 with the primary reviewer. The observed kappa’s were 0.88 for JD and ED and 0.88 for FL and ED. Because of the high interrater agreement, one reviewer (ED) performed the final study selection. In case of uncertainty, the selection was discussed with another reviewer (FL). In the second stage, the full texts of all articles identified as potentially relevant were read by two reviewers (ED and FL). Results were compared and discrepancies among the reviewers were discussed. In case of disagreement, a third reviewer (JD) was consulted.

### Charting the data

The following information was extracted from the included studies: study population, study objective(s), symptoms assessed, symptom assessment method, analytical method and statistical packages used, centrality indices (see Box 1 for more information), results, and the conclusion. When certain information could not be directly retrieved from the article, the authors were contacted. One reviewer (ED) extracted the data. The second reviewer (FL) extracted data of 10% of the papers to check accuracy.

**Table:** Box 1 Centrality indices.

**Centrality indices** Centrality analyses are conducted to measure which variables are most central in the network (i.e., the variables with the strongest connections in the network [21, 30]) Four common measures are as follows: *• Degree centrality*: represents the number of connections of a node [30] *•* *Closeness:* measures the distance between a node and all other nodes in the network [21] *• Betweenness*: measures how often a node lies in the shortest path between every combination of two nodes [21] *• Strength*: is the sum of all edge strengths to or from a node [21] Additional centrality indices exist, which provide insights into different dimensions of centrality	

Symptoms were categorized as somatic, psychological, or undecided. Somatic symptoms were defined as symptoms that are primarily caused by cancer or cancer treatment. Psychological symptoms were defined as symptoms that are primarily aspects of the psychological adjustment to the cancer diagnosis. Certain symptoms have both strong psychological and somatic aspects: these symptoms were labeled as undecided. The classification of symptoms is listed in Appendix B.

### Collating, summarizing, and reporting the results

The associations between somatic and psychological symptoms were extracted. Authors were contacted to retrieve edge weights or partial correlations, if not reported. The measures and reporting of effect sizes varied among the articles. Therefore, for the interpretation of the results we used the authors’ interpretation of the strengths, as well as the absolute or relative edge strength within the network, or the network density (i.e., the number of associations in the network relative to the total number of associations possible). For edge weights and partial correlations, we most often used the relative strength for the interpretation of the associations, for Pearson’s or Spearman’s correlations, coefficients were interpreted as follows: ≤ 0.3 = weak; > 0.3 < 0.7 = moderate; ≥ 0.7 = strong [31]. Additionally, articles were searched for themes that they had in common, to summarize the findings (as will be reported, the themes were the clustering of symptoms, characteristics of symptoms, the associations of symptoms over time, and centrality).

## Results

### Overview of included studies

**Fig. 2:**
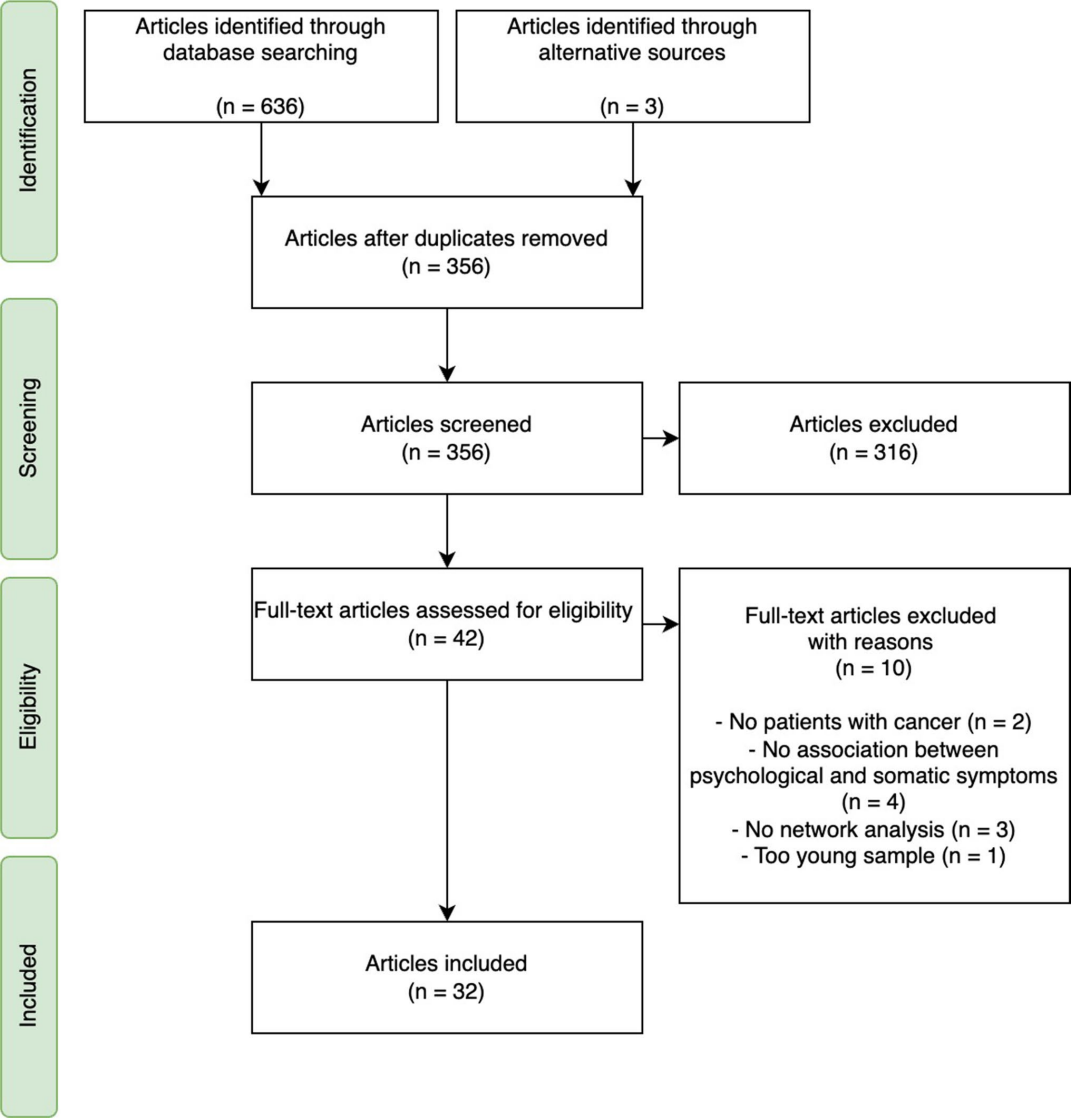
PRISMA flow diagram of the identification and selection process

The initial search yielded 639 articles. After duplicates were removed, 356 unique articles remained. Title and abstract screening excluded 316 articles; 42 articles underwent a second stage of screening based on their full-text. Reviewers reached consensus to include 32 articles in the review. The flow diagram and reasons for exclusion are shown in Fig. 2.

All included articles were published between 2016 and 2023. Over half of the articles were published between 2021 and 2023. Twelve articles included patients during active treatment [3, 32–42], eight articles included patients with cancer who already completed their treatment [2, 16, 18, 43–47], eight studies included patients during and following treatment [21, 48–54], while four articles did not specify treatment status [55–58]. Thirteen articles focused on one cancer type, mostly breast cancer [3, 18, 21, 41, 42, 44, 57]. The other studies investigated multiple myeloma [36], brain [48, 53], head and neck [38, 40], prostate [52], or gastric cancer [33]. Four articles included multiple pre-defined cancer types [32, 34, 35, 45], the remaining thirteen articles did not have specific inclusion criteria regarding cancer type [2, 16, 37, 39, 43, 46, 47, 49–51, 54–56, 58]. Half of the studies had more female than male participants. The mean age ranged from 34 to 68 years. See Table 1 for study characteristics.

**Table 1:** Study characteristics

Authors	Year of publication	Female sex (%)	Mean age (SD)	Sample size	Study aim	Study population	Network analysis (R Package used)
Airaksinen et al	2020	621 (46)^†^	67.68 (6.70)^†^	1337	Examine how depression symptoms are connected before and after the diagnosis of a chronic illness	Cancer patients	Ising models (bootnet)
Bergsneider et al	2023	469 (41.6)	47.6 (13.7)	1128	Use NA and unsupervised clustering to inform precision health-base symptom care for primary brain tumor patients	Primary brain tumor patients	Gaussian Graphical Model networks (qgraph and EGAnet)
Bickel et al	2022	17 (32.1)	62 (15.0)	52	Examine cancer survivors’ care needs for distinct symptoms of depression	Cancer survivors with mild depressive symptoms	Multilevel Vector Autoregression (mlVAR)
Bobevski et al	2022	800 (54.7)	NR	1463	Examine the phenomenology of demoralization and depression symptoms	Cancer patients	Exploratory Graph Analysis (EGAnet)
Bootsma et al	2022	1 (100)	51–60	1	Examine to what extent and how patients gain insight into cancer related fatigue by filling out the EMA and receiving personalized feedback	Patients with severe cancer related fatigue	Contemporaneous and temporal networks (GraphicalVAR)
Cai et al.,.. Wu	2023	761 (100)	48.5 (11.8)	761	Identify age differences and unique associations between cancer-related symptoms in women undergoing chemotherapy	Patients with breast cancer undergoing chemotherapy^‡^	Centrality analysis: strength and closeness (NR)
Cai et al.,.. Yuan	2023	1033 (100)	46.5 (8.1)	1033	Identify subgroups of cancer-related symptoms among young, middle-aged women undergoing chemotherapy	Patients with breast cancer undergoing chemotherapy^‡^	Network analysis (qgraph)
de Rooij et al	2021	834 (63)	61 (15)	1330	Explore the clustering of symptoms among 7 cancer types	Survivors of colorectal, thyroid, breast and ovarian cancer, HL, NHL, or CLL cancer	Regularized partial correlation networks (qgraph, Bootnet, NetworkComparisonTest)
Harnas et al	2021	3 (100)	55.33 (NR)	3	Illustrate how automated individual time series analysis can be applied to personalize CBT for cancer related fatigue	Patients who completed curative treatment with clinical levels of fatigue	Vector Autoregressive Modelling (AutoVAR)
Hartung et al	2019	2050 (51)	58 (11)	4020	Assess the frequency and severity of depressive symptoms in patients with cancer and the general population	Cancer patients	Unregularized Gaussian Graphical Models (qgraph)
Henneghan et al	2021	66 (100)	48.44 (8.73)	66	Visualize symptom-cytokine networks	Breast cancer survivors who ended adjuvant chemotherapy between 6 months and 10 years prior to enrollment	Directed network analysis (igraph)
Henry et al	2018	531 (100)	NR	531	Develop a method termed concordance network clustering for identifying patient communities based on bridge symptoms	Newly diagnosed women with breast cancer (stage I–III)	Concordance networks (qgraph)
Jing et al	2023	613 (100)	49 (9.4)	613	Explore core symptoms of breast cancer patients with endocrine therapy	Patients with breast cancer who expressed estrogen receptors receiving endocrine therapy	Regularized partial correlation network analyses (qgraph)
Kalantari et al	2022	779 (78.9)	56.9 (12.0)	987	Present an analytical model of the relationship among individual symptoms at six time points across two cycles of chemotherapy	Patients with a breast, gastric or lung cancer diagnosis, starting adjuvant or palliative chemotherapy^§^	Ising models (IsingFit)
Kassakowski et al	2016	281 (58)	57.27 (NR)	485	Examine how the Health-related Quality of Life questionnaire is structured	Cancer patients	Gaussian Graphical Models (Huge and qgraph)
Lin et al	2022	45 (26.2)	59.8 (9.9)	172	Examine and visualize the relationship among psychoneurological symptoms over time	Patients with head and neck cancer, receiving intensity modulated radiotherapy	Regularized partial correlation network (qgraph)
Liu et al	2022	180 (58.25)	33.12 (5.87)	309	Examine the interplay between dignity-related distress and quality of life	Young adults (18–39 years old) with any diagnosis of a malignant tumor	LASSO regularized networks (bootnet, qgraph, and networktools)
Murri et al	2023	779 (70)	62.32 (14.54)	1108^¶^	Examine the structure of psychopathology and hostility longitudinally	Cancer outpatients	Gaussian Graphical Models (qgraph and NetworkComparisonTest)
Neijenhuijs et al	2021	701 (68)	60.52 (11.31)	1032	Examine symptom clusters in cancer patients and survivors	Cancer patients or survivors who were treated with curative intent	Undirected networks (tidygraph, ggraph)
Papachristou et al	2019	1023 (77.7)	57.2 (12.4)	1328	Explore the interconnectedness of cancer symptoms using two different models	Cancer patients receiving chemotherapy treatment (Breast, gastrointestinal, gynecological or lung cancer)	Pairwise Markov Random Fields (Isingfit and qgraph)
Poikonen-Saksela et al	2022	487 (100)	52.54(NR)	487	Explore the relations among functions and symptoms and how they change from baseline to 1 year follow-up	Women who had recently completed adjuvant chemotherapy or started endocrine therapy of early breast cancer	Regularized partial correlation networks with Graphical LASSO (qgraph)
Rha and Lee	2021	184 (73.9)	51.89 (9.75)	249	Examine the relationship among symptoms over the course of four cycles of chemotherapy	Patients with a breast, gastric or lung cancer diagnosis, starting adjuvant or palliative chemotherapy^§^	Network analysis (qgraph)
Röttgering et al	2023	95 (37)	46.53 (13.45)	256	Examine the interrelatedness of fatigue, depression, subjective cognitive complaints, brain tumor-related symptoms and health related quality of life	Glioma patients with elevated fatigue	Gaussian Graphical Models based on Spearman’s partial correlation matrices (qgraph, bootnet and NetworkComparisonTest)
Santoso et al	2022	55 (20.8)	65 (8.2)	264	Examine associations between psychoneurological symptoms and biomarkers of stress and inflammation	Head and neck cancer patients; curative intent	Undirected partial correlation networks (Huge and Rags2Ridges)
Schellekens et al	2021	1 (100)	34	1	Explore the usability of providing feedback on symptom network for a patients who sought help for cancer care related fatigue	One patient with severe cancer related fatigue, 2 years after diagnosis	Contemporaneous and temporal networks (GraphicalVAR)
Schellekens et al	2020	264 (77.2)	51.35 (10.62)	342	Contribute to revealing the complex nature of the cross-sectional relationship among symptoms and risk and protective factors	Patients with cancer seeking psychological help	Regularized partial correlation coefficients networks (qgraph)
Sharpley et al	2023	0 (0)	68.07 (6.51)	555	Extend the understanding of the nature of depression in prostate cancer patients	Patients who ended active treatment for prostate cancer	Regularized partial correlation networks (qgraph, igraph and springlass)
Shim et al	2021	92 (36)	62.41 (10.72)	256	Examine patterns of association between cancer-related physical and psychological symptoms and quality of life over time	Gastric cancer patients admitted for curative section surgery	Gaussian Graphical Models (bootnet, NetworkComparisonTest, qgraph)
Van der Stap et al	2022	252 (47.4)	NR	532	Evaluate the feasibility of a Bayesian Network model for predicting the probability of simultaneously occurring symptoms	Patients with advanced cancer	Bayesian Networks (bn learn)
Yang et al	2022	803 (100)	47.25 (10.51)	803	Investigate the interrelationship between fear of recurrence, anxiety and depression	Women with breast cancer	Network analysis with “EBICglasso” method (bootnet and qgraph)
Zeng et al	2023	NR	NR	170	Identify core symptoms in multiple myeloma patients receiving chemotherapy	Patients diagnosed with multiple myeloma undergoing chemotherapy	Network analysis with “EBICglasso” and Pearson’s correlations (qgraph)
Zhu et al	2023	712 (66.85)	65.00 (11.42)	1065	Generate symptom networks of multidimensional symptom experience and explore centrality indices and network density	Cancer survivors who had completed initial treatment	Partial correlation networks (qgraph, mgm, spring and bootnet)

*CLL* chronic lymphocytic leukemia, *EMA* ecological momentary assessment, *HL* Hodgkin lymphoma, *NHL* non-Hodgkin lymphoma, *NA* network analysis, *NR* not reported.

^†^Data two years prior to cancer diagnosis.

^‡,§^Data from the same dataset is used.

^¶^515 for longitudinal analysis.

The studies varied in sample size (range *N* = 1–4020), number of symptoms used in the analysis (range 2–41 symptoms), as well as in the use of single items versus sum scores. Twenty-one articles reported on cross-sectional data, ten reported on longitudinal data, and one article reported on both longitudinal and cross-sectional data. The objective of most articles was to examine the interconnectedness of symptoms in patients with cancer, with a specific focus. The focus entailed for example the development of models that can provide information on symptom networks [21, 54] or the use of individual networks in the treatment of symptoms [16, 43, 46, 47]. All study aims are shown in Table 1. Almost all analyses were conducted in R, with different packages used for the construction of the network. Most articles examined centrality indices such as strength centrality, betweenness and closeness. Other indices such as the stability and accuracy of the networks were also examined, albeit less often. The number of somatic and psychological symptoms that was assessed was most often not equal. In general, more psychological symptoms were included in the models compared to somatic or undecided symptoms.

### Association between somatic and psychological symptoms

All articles reported at least one association between somatic and psychological symptoms in their network(s). The number of associations between somatic and psychological symptoms varied across studies, as well as the strength. The strength of these associations was reported in (the supplementary materials of) eight articles. The authors of 24 articles that did not report on the strength of the associations were contacted. For nine articles, additional information was provided upon request, while for the remaining fifteen articles no additional information was received. Overall, weak associations were reported between somatic and psychological symptoms. Two studies reported that all symptoms within the network were strongly correlated [52, 53]. One study specifically mentioned strong associations between somatic (physical limitations in daily life) and psychological complaints in patients with severe symptoms [49]. The inspection of the available correlation or edge weight matrices showed that cross-domain associations (i.e., between symptoms from the somatic and psychological domain) were often smaller than associations within the same domain. To illustrate, a study among breast cancer patients reported that mood swings and irritability had an edge weight of 0.70, while neither mood swings nor irritability had larger edge weights than 0.13 with any of the somatic items (such as hot flashes or headaches) [3]. Stronger associations within than across domains were observed for both studies examining patients in active treatment and after treatment.

### Clustering of symptoms

Because almost half of the articles did not include (or provide) a correlation or edge weight matrix, we further looked at clusters or communities within the networks. Moreover, we looked at the visualization of the networks to compare the relative strength of the associations. Multiple studies analyzed clusters or communities of symptoms within a network [18, 21, 32, 34, 35, 38, 39, 44, 45, 48, 49, 55, 58], three of which exclusively examined patients after treatment [18, 44, 45]. A number of studies found one or more clusters that consisted of both somatic and psychological symptoms [21, 34, 35, 45, 48, 49, 57, 58]. Examples of these cross-domain clusters are a cluster of anxiety, depression, sleep disturbance, pain and dyspnea [34], or fatigue, pain, emotional symptoms, appetite loss, and dyspnea [45]. More symptom clusters were reported that fall within either the somatic or psychological domain, as compared to cross-domain clusters [18, 32, 34, 35, 39, 48, 55, 58]. Examples are a somatic cluster of faintness, pain on heart, nausea, trouble getting breath, numbness, weakness, and physical problems [39] and a psychological cluster of having no interest, nervousness, feeling lonely, feeling blue, tense and keyed up, scared for no reason, worthlessness, panic, hopelessness, restlessness, thoughts about ending life, fearfulness, and thoughts about death [39]. Overall, the cluster analyses and the visual inspection of networks showed that somatic as well as psychological symptoms were more strongly associated among themselves than with each other. Undecided symptoms such as fatigue were often associated with both somatic and psychological symptoms and were found in both same-domain and cross-domain clusters.

### Characteristics of symptoms

In one of the included articles, Papachristou and colleagues [32] analyzed symptoms of patients receiving chemotherapy in three different ways. They examined symptom occurrence (prevalence of symptoms), symptom severity (importance of symptoms), and symptom distress (influence of symptoms). They reported that the association among symptoms differed depending on what characteristic was used to examine symptoms. Likewise, the corresponding networks showed different associations between somatic and psychological symptoms. Most of the other studies measured severity of symptoms, some measured occurrence and a few used frequency or distress.

Three studies compared the network connectivity of patients with low, moderate, or high severity of symptoms [41, 48, 49]. Neijenhuijs and colleagues [49] examined 26 symptoms in 1330 cancer survivors, of which 60% had completed their treatment; they classified symptoms that were reported by the participants as “no risk for well-being,” “moderate risk for well-being,” or “high risk for well-being.” They observed more connections between self-reported somatic and psychological symptoms in patients with high risk scores, compared to patients with medium-to-high risk scores. In patients with high risk scores, psychological complaints and physical limitations were most central and were connected to nearly all symptoms. Bergsneider and colleagues [48] examined 20 symptoms among 1128 patients receiving treatment, or following treatment for a brain tumor. They found that the subgroup of patients with the highest occurrence and severity of symptoms had a stronger overall interconnectivity among the symptoms within the network, as compared to patients with less severe symptoms. This finding held for the associations between psychological and somatic symptoms as well. Cai and colleagues [41] examined 41 symptoms among 1033 women with breast cancer receiving chemotherapy and analyzed subgroups of patients with different levels of symptom severity. They did not statistically compare the density of the networks, but the network of the severe symptom subgroup showed thicker edges as compared to the moderate or mild symptom subgroups.

### Symptoms over time

Out of the eleven articles that reported on longitudinal data [16, 18, 33–35, 39, 40, 43, 46, 47, 56], nine articles reported on changes in network connectivity over time [16, 18, 33–35, 40, 43, 47, 56]. Despite the differences in time span across the studies (ranging from a couple of weeks to 8 years), and regardless of treatment status (in active treatment or after treatment), the articles reported that symptom clusters remained rather stable over time. Because the authors did not provide an explicit report on whether this was also the case for the associations between somatic and psychological symptoms, the networks were visually inspected. Connections between somatic and psychological symptoms were often visible at multiple time points, while there were also associations that changed from one time point to the next.

Rha and Lee [34] found stable symptom clusters over the course of four cycles of chemotherapy. These entailed the association between anxiety and dyspnea at four out of five time points, for example. Airaksinen and colleagues [56] examined the connectedness of depressive symptoms 4 and 2 years before diagnosis, as well as 2 and 4 years after diagnosis. They found that the connectivity among symptoms had not changed significantly since the time prior to the diagnosis. Moreover, the associations of restless sleep with psychological symptoms of depression (such as sadness or feeling depressed) were present at all time points. In contrast, some studies (e.g., [18, 33]) showed some variation in associations between somatic and psychological symptoms over time. An example is the article from Kalantari and colleagues [35], who used data from the same dataset as [34]. They found a core set of symptoms within the clusters that were consistently present over two cycles of chemotherapy, while the specific associations between somatic and psychological symptoms had changed.

### Central symptoms

Centrality analyses revealed a range of symptoms that were most central within a network. Fatigue was most often reported as (one of) the most central symptom(s), in 16 out of 32 studies [18, 21, 32–35, 38, 40–42, 45, 48, 49, 51, 53, 55, 58], of which seven studies examined patients in active treatment [32–35, 38, 40–42], two studies examined patients following treatment [18, 45], four examined patients during and after treatment [48, 49, 51, 53], and two did not specify treatment status [55, 58]. In contrast, one study among cancer survivors reported that fatigue was among the least central symptoms within the network [2]. No apparent differences were found in the design of this study, as compared to others. Other examples of central symptoms were depression/depressed mood [18, 40, 50, 56], sadness [2, 33, 39, 57], and loss of enjoyment [50, 52]. In longitudinal studies, central symptoms differed across time points. For example, Shim and colleagues [33] found different central symptoms before surgery (shortness of breath and fatigue), 1 week after surgery (fatigue) and 3–6 months after surgery (physical well-being).

### Treatment status

The results regarding associations between somatic and psychological symptoms, the clustering of symptoms, the characteristics of symptoms and symptoms over time were compared between studies that examined patients during, after, or during and after treatment. No substantial differences were found in the results of studies that examined patients in active treatment versus patients after treatment.

## Discussion

This scoping review summarized the evidence on the association between somatic and psychological symptoms in patients with cancer and cancer survivors based on network analysis. Key findings were that (a) stronger associations were reported among psychological symptoms and among somatic symptoms than between somatic and psychological symptoms; (b) the association between somatic and psychological symptoms was stronger in patients experiencing more severe symptoms; (c) the association between somatic and psychological symptoms remained rather stable over time; and (d) different symptoms played a central role in the network, with fatigue being most frequently reported.

### Associations between psychological and somatic symptoms

All studies reported associations between somatic and psychological symptoms. Earlier reviews in patients with cancer also showed connections between somatic and psychological symptoms [59–61]. These reviews focused on specific symptoms and did not limit their search to network analysis. The present study found stronger associations among somatic symptoms and among psychological symptoms than between somatic and psychological symptoms. Previous reviews on symptom clusters in patients with cancer have also identified separate psychological and somatic (gastrointestinal) clusters of symptoms [62, 63]. Therefore, we tentatively conclude that in patients with cancer psychological symptoms and somatic symptoms are more strongly associated among themselves than with each other. This finding may, in part, be due to method variation. Symptoms are likely to be more strongly correlated to symptoms from that same questionnaire as compared to symptoms measured with another questionnaire, because questionnaires are often developed with the intention to have a high internal consistency. Because several articles used one questionnaire for somatic symptoms and another questionnaire for psychological symptoms, it is possible that this explains at least partially the observed pattern of stronger associations among psychological symptoms and among somatic symptoms than between these symptom domains. Therefore, the results of this review should be interpreted with the study objectives and the chosen methods in mind.

### Characteristics of symptoms

One study reported that the associations among somatic and psychological symptoms differed depending on which symptom characteristic was measured (occurrence, severity, or distress) [32]. This is in line with a review on symptoms clusters in patients with cancer, not using network analysis [63]. Regarding symptom severity, three studies found that the association among somatic and psychological symptoms was stronger in patients with more severe symptoms [41, 48, 49]. Because strong association among symptoms might cause more symptoms within the network to be activated in case of an adverse event, this finding is worth examining in future research.

### Association over time

In this review, we found nine longitudinal studies that examined changes in associations over time. These studies reported rather stable associations among somatic and psychological symptoms over time. In contrast, an earlier review on symptom clusters (not using network analysis) found that only over a third of the studies reported relatively stable clusters over time [63], and another review reported low stability in symptom clusters among breast cancer patients over time [64]. It is possible that, in contrast to symptoms clusters in general, the association between psychological and somatic symptoms is rather stable over time. If confirmed in future studies, further research is needed to explain this finding.

### Central symptoms

The majority of studies found that fatigue was one of the most central symptoms within their network. Fatigue has both somatic and psychological aspects; whether somatic aspects or psychological aspects are more prominent may depend on tumor activity and treatment status [65]. Moreover, fatigue is experienced in the majority of patients [15, 46, 54]. Because of its central position, fatigue was proposed as a possible target for interventions [18, 21, 40, 46, 48]. However, other factors than centrality can determine the influence of a symptom on the network [18]. Additionally, the centrality of symptoms is highly dependent on which symptoms are included in the network.

### Treatment status

It is plausible that the nature of symptoms during and after treatment differ, where symptoms during treatment follow the cycles of treatment, and symptoms after treatment may be more persistent in nature. However, no clear differences were found regarding the association between somatic and psychological symptoms, the characteristics of symptoms, symptoms over time, and central symptoms. Possibly, the number of included studies is too small to detect such differences.

### Challenges to study comparison

An overarching challenge to compare study results was the heterogeneity of the studies. Studies differed in design, sample size, symptoms assessed, measurement instruments, and number of time points. This hampered comparing and interpreting the findings. Moreover, the majority of the studies did not report on the associations between somatic and psychological symptoms in detail, as it was not the main focus of these studies. Similarly, while articles visualized networks and often reported on centrality indices, the effect sizes for specific associations could not be retrieved for fifteen articles. Future network analyses would benefit from reporting on the strength, such as (partial) correlation coefficients or edge weights. Burger and colleagues [66] developed reporting standards for network analyses on cross-sectional data.

### Study limitations

This review has several limitations that need to be considered. Firstly, it was not always easy to determine whether symptoms are primarily caused by cancer and its treatment, or primarily by psychological adjustment to the disease. It is therefore important to note that the classification of symptoms is a simplification of the complex interplay between somatic and psychological symptoms. Secondly, studies written in other languages than English were not included.

### Clinical implications

Despite these limitations, this review has some tentative clinical implications. Somatic and psychological symptoms are associated; possibly somatic symptoms activate psychological symptoms, and somatic symptoms activate psychological symptoms. By means of individual networks, it can be examined which somatic and psychological symptoms are particularly connected for a patient (even over time and especially in patients with severe symptoms). This can inform which somatic symptoms can be possible targets for intervention to alleviate psychological symptoms, or which psychological symptoms can be targeted to improve somatic symptoms [67]. This review showed that fatigue is a prominent symptom in half of the studies. This emphasizes the importance of this and other symptoms that can be related to cancer and its treatment, as well as to psychological adjustment. However, more longitudinal studies are necessary to untangle the complex interplay of somatic and psychological symptoms.

### Future directions

This scoping review focused on network analysis to examine symptoms in patients with cancer and cancer survivors. Based on the results from the present study and findings in the field of psychopathology, we argue that future research would benefit from exploring the application of network analysis in the symptom management of patients with cancer further. This study showed the diverse application of this technique. A major benefit of network analysis is that it provides the means to look at the interplay between symptoms, instead of focusing on an underlying disease or disorder. This is especially beneficial when looking at symptoms that can have multiple causes (both disease-related as psychological), as is the case in the symptom experience in patients with cancer. More longitudinal studies are necessary to be able to understand the complex interplay of symptoms better. Longitudinal studies using for example ecological momentary assessment may be a step forward into the understanding of the association between somatic and psychological symptoms. This is because longitudinal studies can show which symptoms are influencing symptoms at later time points, or which symptoms may be easily influenced by other symptoms in the network. Symptoms that impact other symptoms in the network could become targets at interventions, or symptoms to monitor during treatment to decrease a cascading effect of symptoms [68]. Therefore, longitudinal studies add to the knowledge of cross-sectional studies that can only show which symptoms co-occur. Through the limited, yet growing number of available studies, this study highlights the promise of network analysis in the examination—and perhaps even the management—of somatic and psychological symptoms in patients with cancer.

## Conclusions

Although the associations among somatic symptoms and among psychological symptoms were stronger, somatic and psychological symptoms were associated, especially in patients experiencing more severe symptoms. The association between psychological and somatic symptoms remained rather stable over time. Fatigue was one of the most central symptoms, bridging the somatic and psychological domain. This review shed light on the emerging field of research on the association between somatic and psychological symptoms based on network analysis and summarized the major findings. Further longitudinal research using the network approach is needed to unveil the complex interplay of somatic and psychological symptoms in patients with cancer.

## Supplementary Information

Below is the link to the electronic supplementary material.

Supplementary file1 (DOCX 16 KB)

Supplementary file2 (DOCX 63.9 KB)
