# Supplementary material for: Network analysis used to investigate the interplay among somatic and psychological symptoms in patients with cancer and cancer survivors: a scoping review
Source: J Cancer Surviv. 2024 Mar 26;19(4):1198–211. doi: 10.1007/s11764-024-01543-0 (PMC12283480; doi:10.1007/s11764-024-01543-0)
Supplement: Supplementary file 1 — Supplementary file1 (DOCX 16 KB) [file 11764_2024_1543_MOESM1_ESM.docx]

**Network Analysis used to investigate the Interplay among Somatic and Psychological Symptoms in Patients with Cancer: A Scoping Review**

**Journal of Cancer Survivorship**

G. Elise Doppenberg-Smit, MSc^1, 2,4^, Femke Lamers, PhD^1,2^, Myra E. van Linde, MD, PhD^3^, Annemarie M.J. Braamse, PhD^2,4,5^, Mirjam A.G. Sprangers, PhD^2, 4,5^, Aartjan T.F. Beekman, MD, PhD^1, 2^, Henk M.W. Verheul, MD, PhD^6^, Joost Dekker, PhD^1,2,4^

1. Amsterdam UMC, location Vrije Universiteit Amsterdam, Department of Psychiatry, de Boelelaan 1117, Amsterdam, the Netherlands

2. Amsterdam Public Health, Mental Health program, Amsterdam, the Netherlands

3. Amsterdam UMC, location Vrije Universiteit Amsterdam, Department of Medical Oncology, de Boelelaan 1117, Amsterdam, the Netherlands

4. Cancer Centre Amsterdam, Cancer Treatment and Quality of Life, Amsterdam, the Netherlands

5. Amsterdam UMC, location University of Amsterdam, Department of Medical Psychology, Amsterdam, the Netherlands

6. Erasmus MC, Department of Medical Oncology, Dr. Molewaterplein 40, Rotterdam, The Netherlands

**Correspondence**Elise Doppenberg-Smit

g.e.doppenberg@amsterdamumc.nl

+31 6 18 14 88 63

**Cancer:**

"Neoplasms"[Mesh] OR neoplas*[tiab] OR tumor*[tiab] OR tumour*[tiab] OR cancer*[tiab] OR lymphoma*[tiab] OR malignan*[tiab] OR oncolog*[tiab] OR carcinom*[tiab] OR melanom*[tiab]

**AND**

{

**Psychological symptoms:**

"Anxiety"[Mesh] OR "Sadness"[Mesh] OR "Psychological Distress"[Mesh] OR "Fear"[Mesh] OR "Depression"[Mesh] OR "anxiet*"[tiab] OR "panic"[tiab] OR "anxious*"[tiab] OR fear[tiab] OR sadness[tiab] OR depress*[tiab] OR "psychological distress"[tiab] OR "emotional distress"[tiab] OR “emotional symptom*”[tiab] OR "psychological symptom*"[tiab] OR “psychosocial symptom*”[tiab] OR "irritab*"[tiab] OR ("satisfaction"[tiab] NOT "patient satisfaction"[tiab]) OR excitement[tiab] OR upset[tiab] OR annoyed[tiab] OR listlessness[tiab] OR apathy[tiab] OR energetic[tiab] OR enthusiasm[tiab] OR enthusiastic[tiab] OR nervousness[tiab] OR boredom[tiab] OR calmness[tiab] OR worries[tiab] OR worry[tiab] OR worried[tiab]

**OR**

**Physical symptoms:**

"Fatigue"[Mesh] OR "Nausea"[Mesh] OR "Anorexia"[Mesh] OR fatigue*[tiab] OR nausea*[tiab] OR "lack of appetite"[tiab] OR "loss of appetite"[tiab] OR “appetite loss”[tiab] OR anorexia[tiab] OR "lack of concentration"[tiab] OR "concentration problem*"[tiab] OR pain[tiab] OR "shortness of breath"[tiab] OR “dyspnea”[tiab] OR "wheezy"[tiab] OR "somatic symptom*"[tiab] OR "physical symptom*"[tiab] OR "physical health*"[tiab] OR "physical function*"[tiab]

}

**AND**

**Network analysis:**

“network analysis”[tiab] OR “network analyses”[tiab] OR “network approach*”[tiab] OR “network model*”[tiab] OR “network structure*”[tiab] OR “VAR”[tiab] OR “vector autoregressi*”[tiab] OR “vector auto regressi*”[tiab]
