# Supplementary material for: Network analysis used to investigate the interplay among somatic and psychological symptoms in patients with cancer and cancer survivors: a scoping review
Source: J Cancer Surviv. 2024 Mar 26;19(4):1198–211. doi: 10.1007/s11764-024-01543-0 (PMC12283480; doi:10.1007/s11764-024-01543-0)
Supplement: Supplementary file 2 — Supplementary file2 (DOCX 63.9 KB) [file 11764_2024_1543_MOESM2_ESM.docx]

**Network Analysis used to investigate the Interplay among Somatic and Psychological Symptoms in Patients with Cancer: A Scoping Review**

**Journal of Cancer Survivorship**

G. Elise Doppenberg-Smit, MSc^1, 2,4^, Femke Lamers, PhD^1,2^, Myra E. van Linde, MD, PhD^3^, Annemarie M.J. Braamse, PhD^2,4,5^, Mirjam A.G. Sprangers, PhD^2, 4,5^, Aartjan T.F. Beekman, MD, PhD^1, 2^, Henk M.W. Verheul, MD, PhD^6^, Joost Dekker, PhD^1,2,4^

1. Amsterdam UMC, location Vrije Universiteit Amsterdam, Department of Psychiatry, de Boelelaan 1117, Amsterdam, the Netherlands

2. Amsterdam Public Health, Mental Health program, Amsterdam, the Netherlands

3. Amsterdam UMC, location Vrije Universiteit Amsterdam, Department of Medical Oncology, de Boelelaan 1117, Amsterdam, the Netherlands

4. Cancer Centre Amsterdam, Cancer Treatment and Quality of Life, Amsterdam, the Netherlands

5. Amsterdam UMC, location University of Amsterdam, Department of Medical Psychology, Amsterdam, the Netherlands

6. Erasmus MC, Department of Medical Oncology, Dr. Molewaterplein 40, Rotterdam, The Netherlands

**Correspondence**Elise Doppenberg-Smit

g.e.doppenberg@amsterdamumc.nl

+31 6 18 14 88 63

| **Article /** Measure | **Symptom or other variable** | **Psycho-logical** | **Somatic** | **Undecided** | **Other** |
| --- | --- | --- | --- | --- | --- |
| **Papachristou et al., 2019**  **Kalantari et al., 2022** |  |  |  |  |  |
| **Zeng et al., 2022** |  |  |  |  |  |
| MSAS | Difficulty concentrating |  |  | X |  |
|  | Pain |  | X |  |  |
|  | Lack of energy |  |  | X |  |
|  | Cough |  | X |  |  |
|  | Feeling nervous | X |  |  |  |
|  | Dry mouth |  | X |  |  |
|  | Nausea |  | X |  |  |
|  | Feeling drowsy |  |  | X |  |
|  | Numbness or tingling in hands or feet |  | X |  |  |
|  | Difficulty sleeping |  |  | X |  |
|  | Feeling bloated |  | X |  |  |
|  | Problems with urination |  | X |  |  |
|  | Vomiting |  | X |  |  |
|  | Shortness of breath |  | X |  |  |
|  | Diarrhoea |  | X |  |  |
|  | Feeling sad | X |  |  |  |
|  | Sweats |  | X |  |  |
|  | Problems with sexual interest or activity |  |  | X |  |
|  | Worrying | X |  |  |  |
|  | Itching |  | X |  |  |
|  | Lack of appetite |  | X |  |  |
|  | Dizziness |  | X |  |  |
|  | Difficulty swallowing |  | X |  |  |
|  | Feeling irritable | X |  |  |  |
|  | Mouth sore |  | X |  |  |
|  | Weight loss |  | X |  |  |
|  | Hair loss |  | X |  |  |
|  | Constipation |  | X |  |  |
|  | Swelling |  | X |  |  |
|  | Change in way food tastes |  | X |  |  |
|  | I do not look like myself |  |  |  | X |
|  | Changes in skin |  | X |  |  |
| **Papachristou et al., 2019**  **Kalantari et al., 2022** |  |  |  |  |  |
| MSAS - modified | Hot flashes |  | X |  |  |
|  | Chest tightness |  | X |  |  |
|  | Increased appetite |  | X |  |  |
|  | Weight gain |  | X |  |  |
|  | Abdominal cramps |  | X |  |  |
|  | Difficulty breathing |  | X |  |  |
| **Yang et al., 2022**  **Hartung et al., 2019**  **Bobevski et al., 2022**  **Sharpley et al., 2023** |  |  |  |  |  |
| PHQ-9 | Anhedonia | X |  |  |  |
|  | Sad mood | X |  |  |  |
|  | Sleep |  |  | X |  |
|  | Fatigue |  |  | X |  |
|  | Appetite |  | X |  |  |
|  | Guilty | X |  |  |  |
|  | Concentration |  |  | X |  |
|  | Motor problems |  | X |  |  |
|  | Thoughts of death/suicide | X |  |  |  |
| **Yang et al., 2022** |  |  |  |  |  |
| GADS | Nervousness | X |  |  |  |
|  | Uncontrollable worry | X |  |  |  |
|  | Excessive worry | X |  |  |  |
|  | Trouble relaxing | X |  |  |  |
|  | Restlessness | X |  |  |  |
|  | Irritability | X |  |  |  |
|  | Feeling afraid | X |  |  |  |
| FCRS | Afraid of recurrence | X |  |  |  |
|  | Worried/anxious about recurrence | X |  |  |  |
|  | Frequency of recurrence worry | X |  |  |  |
|  | Strong feelings about recurrence | X |  |  |  |
| **Bobevski et al., 2022** |  |  |  |  |  |
| DS-II | Worthless | X |  |  |  |
|  | Pointless | X |  |  |  |
|  | Role in life | X |  |  |  |
|  | Emotional in control | X |  |  |  |
|  | Helpless | X |  |  |  |
|  | Hopeless | X |  |  |  |
|  | Irritable | X |  |  |  |
|  | Coping | X |  |  |  |
|  | Regret | X |  |  |  |
|  | Feeling hurt | X |  |  |  |
|  | Distress | X |  |  |  |
|  | Isolated | X |  |  |  |
|  | Trapped | X |  |  |  |
| DS-II & PHQ-9 | Thoughts of death/suicide | X |  |  |  |
| **Neijenhuijs et al., 2021** |  |  |  |  |  |
| Perceived Stress Scale | Relaxation | X |  |  |  |
| Patient specifieke klachtenlijst | Physical limitations in daily life |  | X |  |  |
| Insomnia Severity Index | Insomnia |  |  | X |  |
| Numeric | Fatigue |  |  | X |  |
|  | Pain |  | X |  |  |
|  | Constipation |  | X |  |  |
|  | Diarrhoea |  | X |  |  |
|  | Lack of appetite |  | X |  |  |
|  | Nausea or vomiting |  | X |  |  |
|  | Shortness of breath |  | X |  |  |
| CHQ | Hearing problems |  | X |  |  |
| Oncokompas expert-based questionnaire | Tinnitus |  | X |  |  |
| HADS | Psychological complaints | X |  |  |  |
| SF-36 ‘cognitive functioning’ | Memory/concentration |  |  | X |  |
| De Jong Gierveld Scale | Social life (loneliness) | X |  |  |  |
| FSFI / IIEF | Intimacy and sexuality |  |  | X |  |
| Body Image Scale | Body image | X |  |  |  |
| EORTC-IN-PATSAT32 | Contact with doctor |  |  |  | X |
| Visual analogue scale | Dedication to work |  |  |  | X |
| Oncokompas expert-based questionnaire | Physical activity |  |  |  | X |
|  | Smoking |  |  |  | X |
| Alcohol 5-shot | Alcohol use |  |  |  | X |
| BMI & short Nutritional Assessment Questionnaire | Body weight |  |  |  | X |
| EORTC QLQ-30 | Financial problems |  |  |  | X |
| Dyadic Adjustment Scale Short Form | Relationship with partner |  |  |  | X |
| Vragenlijst Gezinskenmerken Short Form (Dutch-specific) | Relationship with children |  |  |  | X |
| **Schellekens et al., 2021** |  |  |  |  |  |
| EMA – positive mood | Feeling happy | X |  |  |  |
|  | Feeling enthusiastic | X |  |  |  |
|  | Feeling relaxed | X |  |  |  |
|  | Feeling curious | X |  |  |  |
|  | Feeling content | X |  |  |  |
| EMA – negative | Feeling frustrated | X |  |  |  |
| mood | Feeling agitated | X |  |  |  |
|  | Feeling down | X |  |  |  |
|  | Feeling insecure | X |  |  |  |
|  | Feeling guilty | X |  |  |  |
| EMA | Accepting energy level | X |  |  |  |
| **Schellekens et al., 2021** |  |  |  |  |  |
| **Bootsma et al., 2022** |  |  |  |  |  |
| EMA | Pondering | X |  |  |  |
|  | Allowing rest | X |  |  |  |
|  | Hopeless | X |  |  |  |
|  | Control | X |  |  |  |
|  | Pain |  | X |  |  |
|  | Physical fatigue |  | X |  |  |
|  | Concentration |  |  | X |  |
|  | Motivation | X |  |  |  |
|  | Physically active |  |  |  | X |
|  | Mentally active |  |  |  | X |
| **Bickel et al., 2022** |  |  |  |  |  |
| EMA | Little enjoyment | X |  |  |  |
|  | Feeling down | X |  |  |  |
|  | Fatigue |  |  | X |  |
|  | Feeling inadequate | X |  |  |  |
|  | Lack of concentration |  |  | X |  |
|  | Anxiety | X |  |  |  |
|  | Irritability | X |  |  |  |
|  | Worry | X |  |  |  |
| **Schellekens et al., 2020** |  |  |  |  |  |
| CES-D Depression & Anxiety | Depressed mood | X |  |  |  |
|  | Sleep problems |  |  | X |  |
|  | Concentration problems |  |  | X |  |
|  | Worthlessness | X |  |  |  |
|  | Appetite loss |  | X |  |  |
|  | Anxiety | X |  |  |  |
| CIS-FS | Fatigue |  |  | X |  |
| HDI -Wellbeing | Loss of enjoyment | X |  |  |  |
| RSCL | Physical symptoms |  | X |  |  |
|  |  |  |  |  |  |
| GSBQ – Social roles | Social withdrawal |  |  |  | X |
| ICQ | Helplessness | X |  |  |  |
|  | Acceptance of illness | X |  |  |  |
|  | Perceived benefit of illness |  |  |  | X |
| GAS | Goal disengagement |  |  |  | X |
|  | Goal reengagement |  |  |  | X |
| **Poikonen-Saksela et al., 2022** |  |  |  |  |  |
| EORTC-QLQ-30 | Role functioning |  |  |  | X |
|  | Emotional functioning | X |  |  |  |
|  | Cognitive functioning |  |  | X |  |
|  | Physical functioning |  | X |  |  |
|  | Social functioning |  |  |  | X |
|  | Fatigue |  |  | X |  |
|  | Pain |  | X |  |  |
|  | Insomnia |  |  | X |  |
|  | Financial difficulties |  |  |  | X |
|  | Global health |  |  |  | X |
| EORTC-QLQ-BR23 | Body image | X |  |  |  |
|  | Future perspective | X |  |  |  |
|  | Systemic therapy side effects |  | X |  |  |
|  | Arm symptoms |  | X |  |  |
| BDI-13 | Depression score | X |  |  |  |
| **Bergsneider et al., 2023** |  |  |  |  |  |
| MDASI Brain tumour | Difficulty understanding |  | X |  |  |
|  | Difficulty speaking |  | X |  |  |
|  | Distress | X |  |  |  |
|  | Irritability |  | X |  |  |
|  | Change in bowel patterns |  | X |  |  |
|  | Change in appearance |  |  |  | X |
|  | Weakness |  | X |  |  |
|  | Seizures |  | X |  |  |
|  | Vision impairment |  | X |  |  |
| **Bergsneier et al., 2023** |  |  |  |  |  |
| **Shim et al., 2021** |  |  |  |  |  |
| **Zhu et al., 2023** |  |  |  |  |  |
| MDASI / MDASI | Pain |  | X |  |  |
| Brain tumour | Fatigue |  |  | X |  |
|  | Nausea |  | X |  |  |
|  | Disturbed sleep |  |  | X |  |
|  | Distress | X |  |  |  |
|  | Shortness of breath |  | X |  |  |
|  | Difficulty remembering | X |  |  |  |
|  | Lack of appetite |  | X |  |  |
|  | Drowsiness |  |  | X |  |
|  | Dry mouth |  | X |  |  |
|  | Sadness | X |  |  |  |
|  | Vomiting |  | X |  |  |
|  | Numbness |  | X |  |  |
| **Shim et al, 2021** |  |  |  |  |  |
| FACT-Ga | Physical well-being |  |  | X |  |
|  | Social well-being |  |  |  | X |
|  | Emotional well-being | X |  |  |  |
|  | Functional well-being |  |  |  | X |
|  | Gastric cancer subscale |  | X |  |  |
| **Shim et al., 2021**  **Santoso et al., 2022** |  |  |  |  |  |
| HADS | Anxiety | X |  |  |  |
|  | Depression | X |  |  |  |
| **Santoso et al., 2022** |  |  |  |  |  |
| MFI | Fatigue |  |  | X |  |
| EORTC-QLQ-H&N | Oral pain |  | X |  |  |
| PSQI | Sleep problems |  |  | X |  |
| Saliva sample | Cortisol slope |  |  |  | X |
| Blood sample | CRP |  |  |  | X |
|  | IL-6 |  |  |  | X |
|  | IL-10 |  |  |  | X |
|  | TNF-α |  |  |  | X |
|  | Age |  |  |  | X |
|  | BMI |  |  |  | X |
| **Henneghan et al., 2021** |  |  |  |  |  |
| FACT-PCI subscale | Perceived cognitive impairment |  |  | X |  |
| Loneliness Scale | Loneliness | X |  |  |  |
| PROMIS-Short form | Fatigue |  |  | X |  |
|  | Anxiety | X |  |  |  |
|  | Depression | X |  |  |  |
| PSQI | Sleep quality |  |  | X |  |
| Blood sample | IL-6; TNF-α; GM-CSF; INF-g; IL-2; IL-4; IL-5; IL-7; IL-8, IL-10; IL-12; IL-1b; IL-13 |  |  |  | X |
| **Airaksinen et al., 2020** |  |  |  |  |  |
| CES-D depression | Felt depressed | X |  |  |  |
|  | Everything is an effort |  |  | X |  |
|  | Sleep is restless |  |  | X |  |
|  | Felt alone | X |  |  |  |
|  | Felt sad | X |  |  |  |
|  | Could not get going |  |  | X |  |
|  | Felt happy | X |  |  |  |
|  | Enjoyed life | X |  |  |  |
| **Harnas et al., 2021** |  |  |  |  |  |
| EMA | Fear of recurrence | X |  |  |  |
|  | Fatigue |  |  | X |  |
|  | Avoidance | X |  |  |  |
|  | Social activity |  |  | X |  |
| **Rha & Lee et al., 2021** |  |  |  |  |  |
| Twenty-Symptom list | Anxiety | X |  |  |  |
|  | Depression | X |  |  |  |
|  | Sleep disturbance |  |  | X |  |
|  | Pain |  | X |  |  |
|  | Fatigue |  |  | X |  |
|  | Drowsiness |  |  | X |  |
|  | Difficulty concentrating |  |  | X |  |
|  | Loss of appetite |  | X |  |  |
|  | Taste change |  | X |  |  |
|  | Dyspnoea |  | X |  |  |
|  | Vomiting |  | X |  |  |
|  | Nausea |  | X |  |  |
| **De Rooij et al., 2021** |  |  |  |  |  |
| EORTC QLQ-C30 | Emotional symptoms | X |  |  |  |
|  | Appetite loss |  | X |  |  |
|  | Constipation |  | X |  |  |
|  | Cognitive symptoms | X |  |  |  |
|  | Diarrhoea |  | X |  |  |
|  | Dyspnoea |  | X |  |  |
|  | Fatigue |  |  | X |  |
|  | Nausea/ vomiting |  | X |  |  |
|  | Pain |  | X |  |  |
|  | Sleep problems |  |  | X |  |
| **Röttgering et al., 2023** |  |  |  |  |  |
| CIS | Fatigue severity |  |  | X |  |
|  | Concentration problems |  |  | X |  |
|  | Reduced motivation | X |  |  |  |
|  | Reduced activity level |  |  | X |  |
| CES-D | Depression | X |  |  |  |
| MOS-Cog | Cognitive functioning |  |  | X |  |
| EORTC-QLQ BN-20 | Future uncertainty | X |  |  |  |
|  | Visual disorder |  | X |  |  |
|  | Motor dysfunction |  | X |  |  |
|  | Communication deficit |  | X |  |  |
|  | Headaches |  | X |  |  |
|  | Seizures |  | X |  |  |
|  | Drowsiness |  |  | X |  |
| **Röttgering et al., 2023**  **Kossakowski et al., 2016**^†^  **Lui et al., 2022** |  |  |  |  |  |
| SF-36 | Emotional role limitations | X |  |  |  |
|  | Physical role limitations |  | X |  |  |
|  | Mental health | X |  |  |  |
|  | Physical functioning |  | X |  |  |
|  | Bodily pain |  | X |  |  |
|  | Vitality |  |  | X |  |
|  | Social functioning |  |  |  | X |
|  | General health |  |  |  | X |
| **Lui et al., 2022** |  |  |  |  |  |
| PDQ | Developmental distress |  |  |  | X |
|  | Spiritual distress | X |  |  |  |
|  | Limited social support |  |  |  | X |
|  | Emotional distress | X |  |  |  |
|  | Symptom distress |  |  | X |  |
| **Murri et al., 2023** |  |  |  |  |  |
| BSI | No interest | X |  |  |  |
|  | Nervousness | X |  |  |  |
|  | Loneliness | X |  |  |  |
|  | Tense and keyed up | X |  |  |  |
|  | Feeling blue | X |  |  |  |
|  | Scared for no reason | X |  |  |  |
|  | Worthless | X |  |  |  |
|  | Panic | X |  |  |  |
|  | Hopelessness | X |  |  |  |
|  | Restlessness | X |  |  |  |
|  | Suicidal thoughts | X |  |  |  |
|  | Death thoughts | X |  |  |  |
|  | Fearfulness | X |  |  |  |
|  | Irritated | X |  |  |  |
|  | Temper outbursts | X |  |  |  |
|  | Urges harming | X |  |  |  |
|  | Urges breaking | X |  |  |  |
|  | Frequent arguments | X |  |  |  |
|  | Practical problems |  |  |  | X |
|  | Family problems |  |  |  | X |
|  | Faintness |  | X |  |  |
|  | Pains on heart |  | X |  |  |
|  | Nausea |  | X |  |  |
|  | Trouble getting breath |  | X |  |  |
|  | Numbness |  | X |  |  |
|  | Weakness |  | X |  |  |
|  | Physical problems |  | X |  |  |
| **Lin et al., 2022** |  |  |  |  |  |
| MFI | Fatigue |  |  | X |  |
| CTC AE | Pain |  | X |  |  |
| PSQI | Sleep disturbance |  |  | X |  |
| PHQ-8 | Depression | X |  |  |  |
| CTC AE | Cognitive dysfunction | X |  |  |  |
| **Jing et al., 2023** |  |  |  |  |  |
| Fact-ES | Mood swings | X |  |  |  |
|  | Irritable | X |  |  |  |
|  | Lost interest in sex |  |  | X |  |
|  | Hot flashes |  | X |  |  |
|  | Cold sweats |  | X |  |  |
|  | Night sweats |  | X |  |  |
|  | Vaginal discharge |  | X |  |  |
|  | Vaginal itching/irritation |  | X |  |  |
|  | Vaginal dryness |  | X |  |  |
|  | Pain or discomfort with intercourse |  | X |  |  |
|  | Breast sensitivity/tenderness |  | X |  |  |
|  | Weight gain |  | X |  |  |
|  | Dizziness |  | X |  |  |
|  | Headaches |  | X |  |  |
|  | Pain in joints |  | X |  |  |
| **Van der Stap et al., 2022** |  |  |  |  |  |
| USD | Anxiety | X |  |  |  |
|  | Depressed mood | X |  |  |  |
|  | Fatigue |  |  | X |  |
|  | Sleep problems |  |  | X |  |
|  | Pain |  | X |  |  |
|  | Dry mouth |  | X |  |  |
|  | Dysphagia |  | X |  |  |
|  | Lack of appetite |  | X |  |  |
|  | Constipation |  | X |  |  |
|  | Nausea |  | X |  |  |
|  | Shortness of breath |  | X |  |  |
| **Cai … Yuan, 2023**^†^ |  |  |  |  |  |
| **Cai … Wu, 2023** |  |  |  |  |  |
| PROMIS-57 | Anxiety | X |  |  |  |
|  | Depression | X |  |  |  |
|  | Fatigue |  |  | X |  |
|  | Sleep disturbance |  |  | X |  |
|  | Pain interference |  | X |  |  |
|  | Pain intensity |  | X |  |  |
| **Henry et al., 2018** |  |  |  |  |  |
| BCTPSS - adjusted | Fatigue |  |  | X |  |
|  | Mouth ulcers |  | X |  |  |
|  | Restless sleep |  |  | X |  |
|  | Sleeping too much |  |  | X |  |
|  | Nervousness or shakiness inside |  |  | X |  |
|  | Mood changes | X |  |  |  |
|  | Feeling depressed | X |  |  |  |
|  | Light-headedness when standing up |  | X |  |  |
|  | Faintness or dizziness at rest |  | X |  |  |
|  | Headaches |  | X |  |  |
|  | Swelling of ankles or feet |  | X |  |  |
|  | Diarrhoea |  | X |  |  |
|  | Nausea |  | X |  |  |
|  | Constipation |  | X |  |  |
|  | Abdominal pain/cramps |  | X |  |  |
|  | Vaginal dryness |  | X |  |  |
|  | Muscle pain/ache/cramp |  | X |  |  |
|  | Weight gain |  | X |  |  |
|  | Weight loss |  | X |  |  |
|  | General aches and pains |  | X |  |  |
|  | Hot flashes |  | X |  |  |
|  | Joint pains |  | X |  |  |
|  | Night sweats |  | X |  |  |
|  | Aches in back of neck and skull |  | X |  |  |
|  | Forgetfulness | X |  |  |  |
|  | Difficulty concentrating |  |  | X |  |
|  | Increased appetite |  | X |  |  |
|  | Short temper | X |  |  |  |
|  | Decreased efficiency |  |  | X |  |
|  | Loss of interest in work/activities | X |  |  |  |
|  | Lowered work performance |  |  | X |  |
|  | Blind spots, fuzzy vision |  | X |  |  |
|  | Breast sensitivity/tenderness |  | X |  |  |
|  | Avoidance of social affairs |  |  |  | X |
|  | Cold sweats |  | X |  |  |
|  | Decreased appetite |  | X |  |  |
|  | Feeling of suffocation |  | X |  |  |
|  | Difficulty healing |  |  |  | X |
|  | Bloating |  | X |  |  |

^†^ Individual items were also analysed separately, in addition to the sum or domain scores.

Note. Somatic symptoms: primarily caused by the tumor or the cancer treatment. Psychological symptoms: primarily aspects of psychological adjustment to the disease and its treatment. Undecided symptoms: related to the tumor/treatment as well as psychological adjustment. ‘Other’ refers to other non-symptom variables in the network.

Abbreviations: BCPTSS = Breast Cancer Prevention Trial Symptom Scale; BDI-13 = Beck’s Depression Inventory-13; BSI = Brief Symptom Inventory; CES-D = Center for epidemiologic studies Depression Scale; CHQ = Caron hearing questionnaire; CIS(-FS) = Checklist Individual Strength (Fatigue Severity); CTC AE = Patient Reported Outcomes version of the Common Terminology Criteria for Adverse Events; DS-II = Demoralisation Scale-II; EMA = Ecological Momentary Assessment; EORTC-QLQ-30 = European Organization for the Research and Treatment of Cancer Quality of Life Questionnaire-30; EORTC-QLQ BN-20 = European Organization for Research and Treatment of Cancer brain tumour module; EORTC-QLQ H&N = European Organization for the Research and Treatment of Cancer Quality of Life Questionnaire, Head and Neck Cancer specific module; FACT-ES = Functional Assessment of Cancer Therapy- Endocrine Subscale; FACT-Ga = Functional Assessment of Cancer Therapy – Gastric; FACT-PCI = Functional Assessment of Cancer Therapy - Perceived Cognitive Impairment Scale; FCRS = Fear of Cancer Recurrence Scale; FSFI = Female Sexual function index; GADS = General Anxiety Disorder Scale; GAS = Goal Adjustment Scale; GSBQ – Social roles = Dutch Groningen Social Behaviour Questionnaire – Social roles subscale; HADS = Hospital Anxiety and Depression Scale; HDI-wellbeing = Dutch Health and Disease Inventory – Wellbeing subscale; ICQ = Dutch Illness Cognitions Questionnaire; IIEF = International Index of Erectile Function; MDASI = MD Anderson Symptom inventory; MFI = Multidimensional Fatigue Inventory; MOS-Cog = Medical Outcomes Study Cognitive Functioning Scale; MSAS = The Memorial Symptom Assessment Scale; PDQ = Patient Dignity Questionnaire; PHQ = Patient Health Questionnaire; PROMIS = Patient-Reported Outcomes Measurement Information System; PSQI = Pittsburg Sleep Quality Index; RSCL = Rotterdam Symptom Checklist; SF-36 = Short Form Health Survey-36; USD = Utrecht Symptom Diary.
